# Progressive Purkinje Cell Degeneration in *tambaleante* Mutant Mice Is a Consequence of a Missense Mutation in HERC1 E3 Ubiquitin Ligase

**DOI:** 10.1371/journal.pgen.1000784

**Published:** 2009-12-24

**Authors:** Tomoji Mashimo, Ouadah Hadjebi, Fabiola Amair-Pinedo, Toshiko Tsurumi, Francina Langa, Tadao Serikawa, Constantino Sotelo, Jean-Louis Guénet, Jose Luis Rosa

**Affiliations:** 1 Département de Biologie du Développement, Institut Pasteur, Paris, France; 2 Institute of Laboratory Animals, Graduate School of Medicine, Kyoto University, Kyoto, Japan; 3 Departament de Ciències Fisiològiques II, IDIBELL, Campus de Bellvitge, Universitat de Barcelona, L'Hospitalet de Llobregat, Barcelona, Spain; 4 Cátedra de Neurobiología del Desarrollo “Remedios Caro Almela”, Instituto de Neurociencias de Alicante, Universidad Miguel Hernández y CSIC, Alicante, Spain; University of Minnesota, United States of America

## Abstract

The *HERC* gene family encodes proteins with two characteristic domains: HECT and RCC1-like. Proteins with HECT domains have been described to function as ubiquitin ligases, and those that contain RCC1-like domains have been reported to function as GTPases regulators. These two activities are essential in a number of important cellular processes such as cell cycle, cell signaling, and membrane trafficking. Mutations affecting these domains have been found associated with retinitis pigmentosa, amyotrophic lateral sclerosis, and cancer. In humans, six *HERC* genes have been reported which encode two subgroups of HERC proteins: large (HERC1-2) and small (HERC3-6). The giant HERC1 protein was the first to be identified. It has been involved in membrane trafficking and cell proliferation/growth through its interactions with clathrin, M2-pyruvate kinase, and TSC2 proteins. Mutations affecting other members of the HERC family have been found to be associated with sterility and growth retardation. Here, we report the characterization of a recessive mutation named *tambaleante*, which causes progressive Purkinje cell degeneration leading to severe ataxia with reduced growth and lifespan in homozygous mice aged over two months. We mapped this mutation in mouse chromosome 9 and then performed positional cloning. We found a G⇔A transition at position 1448, causing a Gly to Glu substitution (Gly483Glu) in the highly conserved N-terminal RCC1-like domain of the HERC1 protein. Successful transgenic rescue, with either a mouse BAC containing the normal copy of *Herc1* or with the human *HERC1* cDNA, validated our findings. Histological and biochemical studies revealed extensive autophagy associated with an increase of the mutant protein level and a decrease of mTOR activity. Our observations concerning this first mutation in the *Herc1* gene contribute to the functional annotation of the encoded E3 ubiquitin ligase and underline the crucial and unexpected role of this protein in Purkinje cell physiology.

## Author Summary

The cerebellum is a coordination center whose function is to fine-tune vertebrates' gait and balance; and for this reason, alterations or damage affecting this structure result in a complex syndrome, called ataxia, with neurological signs that are easily recognized. In the mouse, many mutations producing ataxia have been identified and characterized. They have contributed to a better understanding of the genetics of cerebellum development, physiology, and pathology. The present study identifies the recessive allele responsible for the progressive and massive degeneration of the Purkinje cells observed in mutant mice previously named *tambaleante*. The mutation leads to a single amino acid substitution in a highly conserved domain (RCC1-like) of the giant protein HERC1. This protein belongs to the families HECT (E3 ubiquitin ligases) and RCC1 (GTPases regulators). While a variety of mutations have been reported in several members of these families, leading to sterility, growth retardation, retinitis pigmentosa, amyotrophic lateral sclerosis, or cancer, no mutation had ever been reported so far in the mouse *Herc1* gene. This report demonstrates the crucial and unexpected role of HERC1 in Purkinje cell physiology that could be considered helpful in the development of new therapeutic strategies for neurodegenerative disorders.

## Introduction

The cerebellum plays the role of a coordination centre, integrating peripheral sensory information on movement and position of the body parts to fine-tune gait and balance. Structural or functional alterations of this part of the central nervous system result in a complex syndrome, called ataxia, which is characterized by neurological signs that are clinically obvious in most species including the mouse. Many such mutations, either of spontaneous origin or resulting from strategies of genetic engineering performed *in vitro*, have been studied in detail in this species that, synergistically with human studies, have allowed advancement of our understanding of the developmental mechanisms generating the uniquely complex mature cerebellum.

In this publication, we report the positional cloning of an autosomal recessive mouse mutation, called *tambaleante* (symbol *tbl*; meaning staggering in Spanish), which is precisely characterized by a severe ataxic syndrome [1],[2]. Mice homozygous for this mutation (*tbl/tbl*) exhibit an unstable gait, abnormal hindlimb posture and tremor. All these phenotypic characteristics correlate with a progressive degeneration of Purkinje cells (PCs) starting by two months of age. *tbl* mice thus represent a model of recessively inherited ataxia with progressive neurodegeneration of PCs. Using a combination of genetic, histological and biochemical approaches, we have been able to characterize the pathology of this mutation that we could relate to a mutation in the gene encoding the E3 ubiquitin ligase HERC1.

## Results

### Characterization of the *tambaleante* mutation

**Figure 1:**
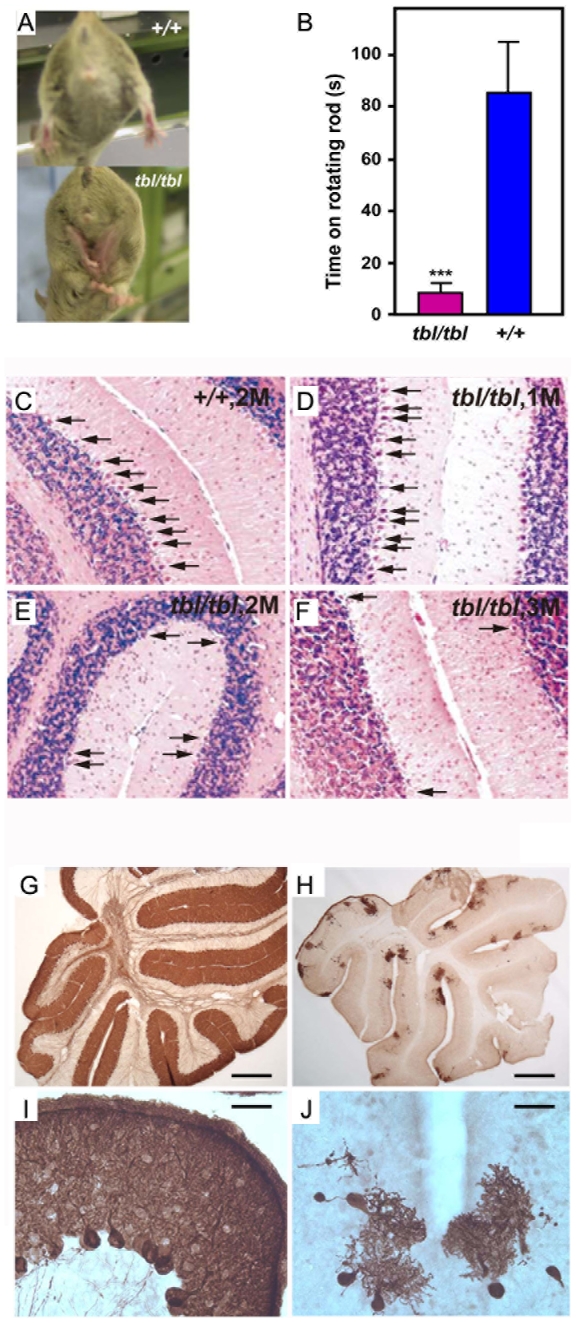
Characteristics of *tambaleante* mice. Hind limbs clasping reflex (A) and rotarod performance (B) of *tambaleante* (*tbl/tbl*) and control (*+/+*) mice. Data show mean±s.d. *** p<0.001 (C) H&E stained sections of the cerebellum of a +/+ control mouse aged two months. (D–F) H&E sections of the cerebellum of *Herc1^tbl^/Herc1^tbl^* mice aged respectively of 1, 2, and 3 months (M), exhibiting Purkinje cell degeneration. Anti-calbindin D28-k staining of parasagittal sections of a normal (G,I) and *tambaleante* (H,J) mouse cortex aged 4 months. The cortex of the mutant mouse is almost completely depleted of PCs. Scale bars: (G,H) 500 µm; (I,J) 25 µm.

**Figure 2:**
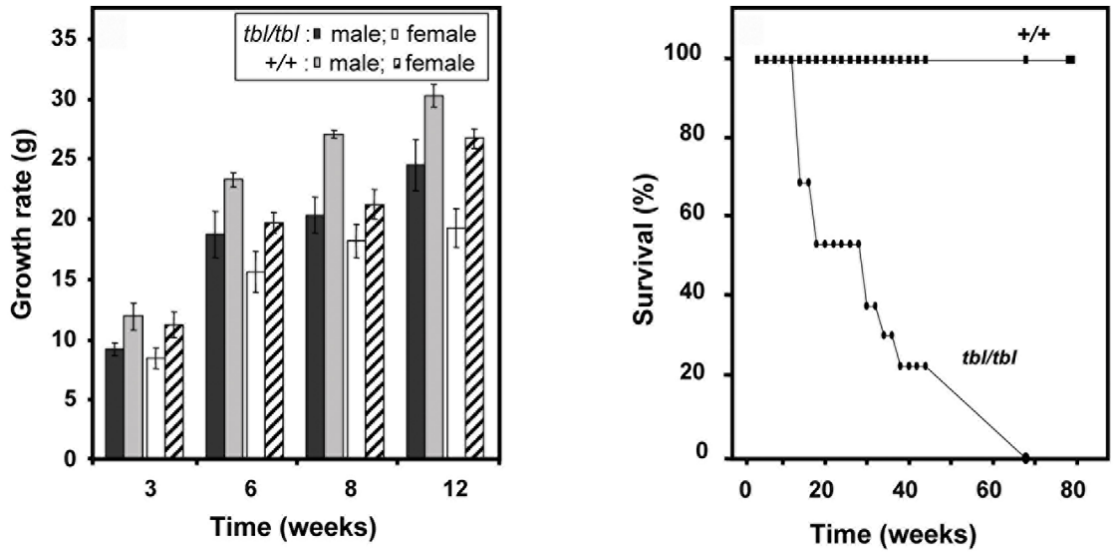
Growth and lifespan of *tambaleante* mice and control. Graphs of growth (left) and survival (right) from mice *tambaleante* (*tbl/tbl*) and mice control (*+/+*). Growth was analyzed in mice (n>9) aged 3–12 months of age. Data show mean±s.d.

The *tambaleante* (*tbl*) mutation arose spontaneously in the DW/JPas inbred substrain, at the Institut Pasteur, and appeared to be inherited as an autosomal recessive condition with complete penetrance. The most remarkable phenotypic feature of homozygous (*tbl/tbl*) mice was an unstable gait, with abnormal hind limb-clasping reflex, which became really obvious from two months of age and worsened with time (Figure 1A and Video S1). To quantify these observations, we performed rotarod assays with these animals. Figure 1B shows that *tbl* animals stayed less time on the rotarod without falling. To visualize the progressive degeneration of PC, we performed an analysis of cerebellum sections stained with haematoxylin and eosin (H&E). In Figure 1C–1F, we can observe the great loss of PC between 1–3 months in *tbl* animals. Immunostaining using anti-calbindin D28-k antibodies (Figure 1G–1J) of parasagital sections of mouse cortex of 4 month old shows that *tbl* mice is almost completely depleted of PC. Compared to their normal littermates, *tbl/tbl* homozygotes were smaller in size. Growth curves showed that the weight of the mutant animals was significantly and constantly lower than the weight of controls, varying from 15 to 30% according to age and gender (Figure 2). Mutant animals also showed a lower survival rate since less than 40 percent of the latter survived longer than 40 weeks on the original DW background (Figure 2). Both sexes appeared to be fertile although poor breeders.

### Mapping and identification of the *tambaleante* mutation

**Figure 3:**
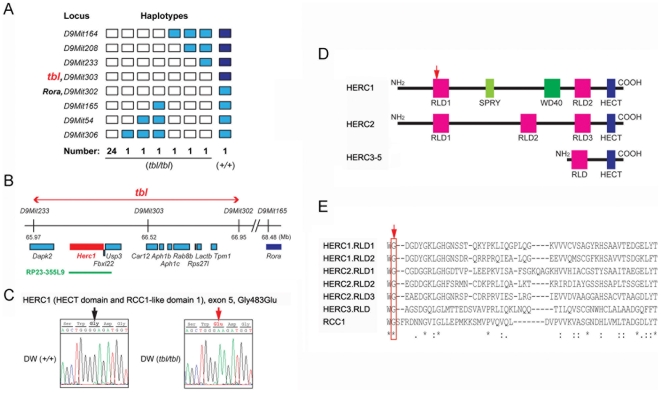
Molecular genetics of the *Herc1^tbl^* locus. (A) Genotyping 30 *tbl/tbl*–F2 mice (60 meiotic events) of an intersubspecific cross allowed to map the *tbl* locus to mouse chromosome 9 between *D9Mit233* and *D9Mit165*. White rectangles symbolize homozygosity for the DW genotype, blue rectangles symbolize heterozygosity, and deep blue homozygosity for MBT genotype. A single mouse, with a crossover between *D9Mit302* and *D9Mit303* and a +/+ genotype at the *tbl* locus, allowed us to eliminate the *Rora* locus (where the mutation *staggerer* occurred) as a candidate. (B) The *tbl* candidate region contains eleven genes. BAC clone RP23-355L9 (shown as a green line) was used for transgenic rescue. (C) Sequence analysis of the cDNA from *Herc1^tbl^*/*Herc1^tbl^* and +/+ DW mice showed a point mutation in exon 5, resulting in a Gly⇔Glu substitution. This missense mutation is located in the RCC1-like domain (RLD) 1 of the HERC1 protein (arrow in D) and changes a highly preserved glycine in the HERC and RCC1 family of proteins (arrow in E; see also [11]). Mouse HERC family and characteristic domains are also shown (D; see text for details).

**Figure 4:**
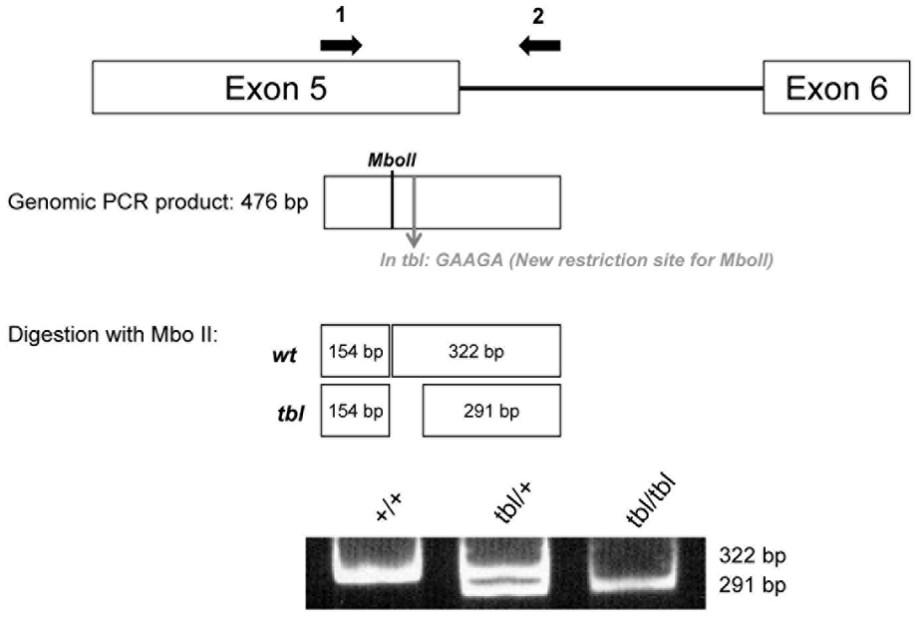
Genotyping analysis of *tbl* mutation. The *tbl* mutation generates a new restriction site (GAAGA) for the *Mbo*II enzyme. PCR amplification of genomic DNA samples of wild-type (*+*) or *Herc1^tbl^* haplotypes with specific primers followed by digestion of the amplification products with the *Mbo*II enzyme allows identifying a haplotype specific pattern by PAGE.

Genotyping 30 F2 mutant offspring (60 meiotic events) of an inter-subspecific cross between DW-*tbl/tbl* males and wild type (+/+) females of the inbred strain MBT/Pas [3], allowed us to assign the locus for *tbl* to chromosome 9, within a 1.7 cM interval flanked by markers *D9Mit233* and *D9Mit165* (Figure 3A and 3B). Although this interval encompassed the locus of the staggerer (*Rora^sg^*) mutation, which occurred in the gene encoding RAR-related orphan receptor alpha and is also characterized by a severe cell-autonomous defect of Purkinje cell [4], we could eliminate this gene as causative of the *tambaleante* phenotype through the finding and characterization of a recombination event between the loci for *tbl* and the one of *Rora* (Figure 3A). In addition, a complementation test performed by mating *tbl/tbl* mice to +/*Rora^sg^* mice and yielding exclusively normal offspring confirmed non allelism of the two mutations (data not shown). Finally, the *tbl* candidate region was reduced to a genomic segment of 0.6 cM (∼0.98 Mb) between *D9Mit233* (65.97 Mb) and *D9Mit302* (66.95 Mb), which contains eleven known genes as indicated in the *Ensembl* sequence database (http://www.ensembl.org/Mus_musculus) (Figure 3A and 3B). Among the eleven genes that were identified in the interval, three candidates (*Herc1*; *Usp3* and *Rab8b*) appeared top ranked considering their expression profile and the known or putative functions of the encoded proteins (Figure 3B). Among these three candidates, *Herc1* seemed to be the most likely one considering its large size and its sequence homology with *Herc2*, a locus where mutations that have phenotypes similar to *tambaleante* have already been reported [5],[6]. Sequence analysis of the ∼15 kb cDNA corresponding to this gene and comparison with the sequences of the co-isogenic strain DW, allowed to identify a single nucleotide difference (a G1448A transition at ENSMUST00000042824) between the *tbl* and the normal (+) haplotype that resulted in a Gly483Glu substitution (Figure 3C). Sequence comparison with several other unrelated inbred strains confirmed that this sequence alteration was recent and unique to the mutant haplotype. The G⇔A transition in the *tambaleante* haplotype generated a new restriction site for the *MboII* enzyme that allowed us to design a PCR assay, helpful for the diagnostic of *tbl* haplotype by discriminating +/+ from *+/tbl* or *tbl/tbl* (Figure 4).

### Rescue of *tambaleante* phenotype

**Figure 5:**
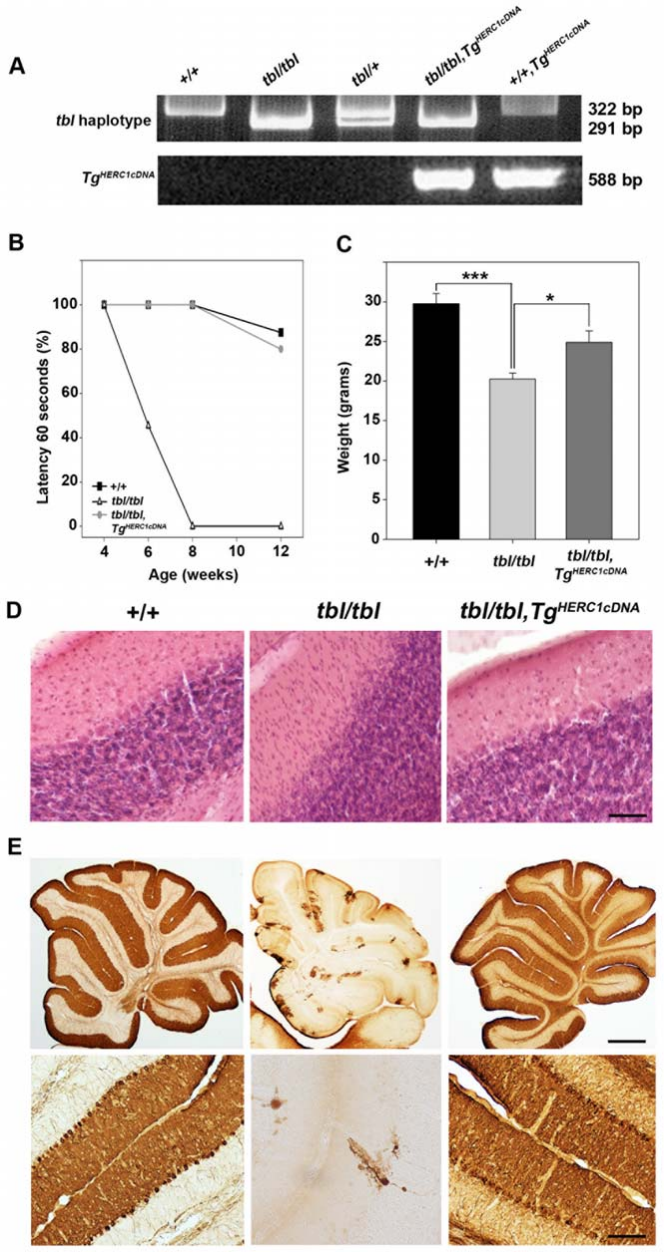
Rescue of the *tambaleante* phenotype. (A) Genotyping analysis by PCR of genomic DNA to identify transgenic and *tambaleante* animals. (B) Motor coordination was tested in wild-type (+/+), homozygous (*tbl/tbl*), and homozygous with transgenic copies (*tbl/tbl;Tg^HERC1cDNA^*) animals (n = 5–9). Testing began at 4 weeks of age and was conducted until week 12 to follow the progression on each phenotype. The animals were put on the rotarod until the latency to fall off reached the total time of 60 s and the percentatge (%) of animals that stayed during this time was represented. (C) Weight chart of these animals (n = 5–9) aged 3–6 months of age. Data show mean±s.d. *** p<0.001, * p<0.05. Staining with H&E (D) and immunostaining with anti-calbindin D28k antibodies (E) of cerebellum sections of these animals. Scale bars: (D,E lower pictures) 25µm; (E upper pictures) 500µm. See Materials and Methods for detailed protocols.

To ascertain that the Gly483Glu substitution identified in *Herc1* was really causative of the abnormal phenotype observed in *tbl/tbl* mice, we decided to attempt the rescue of the *tambaleante* phenotype by crossing *tbl/tbl* mice with transgenic mice expressing a normal copy of the *Herc1* gene. We used two strategies to generate such transgenic mice: in the first case we used the mouse BAC clone RP23-355L9 (∼160 kb - encompassing the *Herc1* locus and a gene encoding a leucine-rich repeat protein 22 (*Fbxl22*) that is not expressed in the brain [7] (Figure 3B)); then we used the human cDNA of *HERC1* (96% amino acid identity with mouse) [8]. BAC transgenic (*Tg^RP23-355L9^/+*) mice were crossed with heterozygous (*+/tbl*) mice, then the F1 mice were mated with heterozygous (*+/tbl*) mice to obtain *tbl* homozygous mice with the transgenic copies (*tbl/tbl*; *Tg^RP23-355L9^/+*). We found that the phenotype of these mice was greatly improved since none of the animals exhibited the phenotypic characteristics of *tambaleante* mutants during the period they were observed (as an example see footprint experiments in Figure S1). By 3 months of age, PCs in these transgenic mice remained normal at least in number and size, indicating complete phenotypic rescue (Figure S1). Complete transgenic rescue was also achieved with the other transgenic strain generated from the human *HERC1* cDNA [6] in pCI-*neo* (*Tg^HERC1cDNA^*) (Figure S2). The complete phenotypic rescue was also analyzed by weight, rotarod performance, cerebellar staining with H&E and immunohistochemistry with anti-calbindin D28-k antibodies (Figure 5). No differences in the parameters analyzed were observed between wild-type animals and transgenic mice. From these results we considered that the pathology in *tambaleante* mice is indeed a direct consequence of the molecular defect in *Herc1*. For this reason, we use *Herc1^tbl^* as an official symbol for the *Herc1* mutant allele.

### Expression of the *Herc1* gene and functional analysis

**Figure 6:**
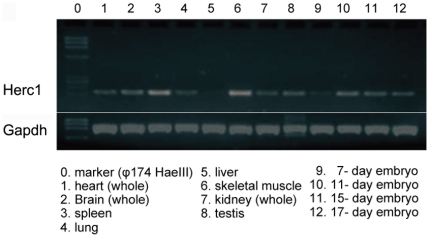
*Herc1* gene expression in mice. *Herc1* is widely expressed in various tissues as indicated by RT–PCR. *Gapdh* expression was used as control.

Analysis of the *Herc1* gene expression by Northern blotting has been previously reported and revealed ubiquitous expression in mammalian tissues although at very low levels in the liver [8]. We have confirmed these data by RT-PCR in mouse tissues using specific primers for *Herc1* (Figure 6). *In situ* hybridization of brain sections also confirmed a pattern similar to that shown in the Allen Brain Atlas [9, and data not shown].

**Figure 7:**
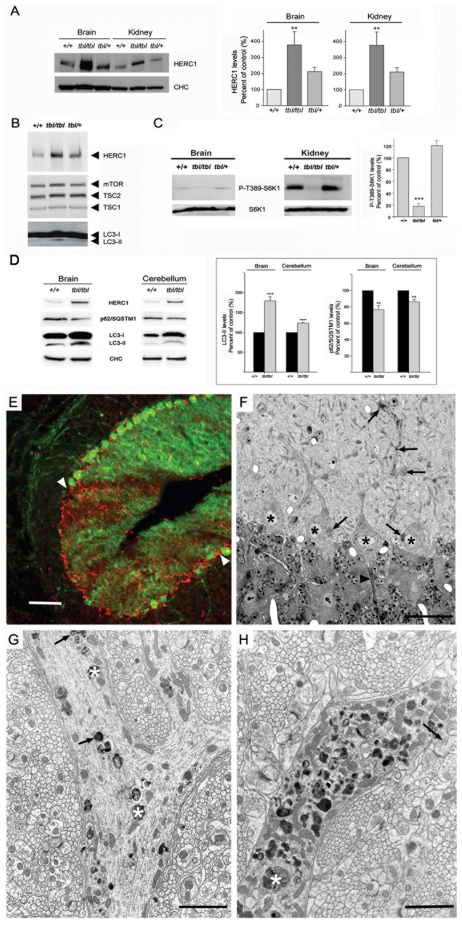
*Herc1* expression and functional analysis. Brain (A–D), kidney (A,C) and cerebellum (D) homogenates from wild-type (*+/+*), heterozygous (*+/tbl*) and homozygous (*tbl/tbl*) mice were analyzed by Western-blot using specific antibodies against the indicated proteins. HERC1 (in brain and in kidney), P-T389-S6K1 (in kidney), p62/SQSTM1 and LC3-II (in brain and in cerebellum) levels were quantified (n = 4–9) and expressed as the mean±s.d. of percentage of respective control. ** p<0.01,*** p<0.001. (E) Parasagittal section of a 2-month-old *tbl/tbl* cerebellum double immunostained with anti-calbindin D28k antibodies to visualize PC (green) and anti-LAMP-1 (red) to identify lysosomes and autophagosomes. The arrowheads point to an area of the cerebellar cortex almost devoid of PC. In this area, LAMP-1 positive puncta are numerous, testifying for the degeneration of PC. (F) Parasagittal 1µm-thick plastic section stained with toluidine blue, illustrating four PC somata (asterisks). The arrows point to dense cytoplasmic inclusions accumulated in the cell bodies and proximal dendrites, particularly at their branching points. The arrowhead points to the first Ranvier node of a PC axon initial segment, which looks normal. (G,H) Electron-micrographs made from the same mouse showing two profiles of the proximal dendritic compartment. The one in (G) illustrates a branching zone in an early stage of the autophagic process. The dendrite contains some vacuoles bound by a smooth double membrane (arrows), enclosing whorls of membrane-like elements, and dense debris (asterisks) suspended in an electronlucent matrix. The other dendritic profile (H) corresponds to a more advanced stage in autophagy, characterized by the occurrence of extremely numerous single membrane bound vacuoles corresponding to autolysosomes, some of them of large size (asterisk). The arrow points to a giant spine emerging from the dendrite and postsynaptic to several parallel fibers varicosities. *Scale bars*: the bar is equal to 100 µm in (E), 24 µm in (F), 1.2 µm in (G), and 1.5 µm in (H).

The HERC proteins have a HECT (Homologous to E6-AP COOH-terminus) domain and at least one domain related with RCC1 (Regulator of Chromosome Condensation 1). HECT domains are involved in the transfer of ubiquitin or ubiquitin-like proteins to target substrates. RCC1-like domains (RLD) seem more versatile and may have a role in guanine nucleotide exchange on small GTP-binding proteins, in enzyme inhibition and in interaction with proteins and lipids. Proteins containing some of these domains are important regulators of cellular processes such as cell cycle, cell signalling and membrane trafficking. HERC1 protein was the first protein of this family to be identified. It contains one HECT domain, two RLD (RLD1 and RLD2), seven WD40 repeats and one SPRY domain (Figure 3D and 3E) [10]–[12]. The Gly483Glu substitution found in *tambaleante* mice is located within the highly conserved N-terminal RCC1-like domain (RLD1) of the HERC1 protein and presumably alters its structure and function (Figure 3D and 3E) [10]. To check whether *Herc1^tbl^* mutation affected the HERC1 protein levels, we performed Western-blot analysis using anti-HERC1 antibodies with samples from brain, cerebellum and kidney of mice older than 3 months. Surprisingly, we observed a significant increase in the amount of HERC1 protein in mutant mice, suggesting a possible increase of its stability (Figure 7A and 7D). No changes were observed with any other protein such as clathrin heavy chain (CHC) that was used as loading control (Figure 7A). Similar results were also found in skeletal muscle (not shown).

HERC1 has been previously reported to interact with TSC2 protein. *TSC1* and *TSC2* are tumour-suppressor genes that are mutated in the tumour syndrome TSC (tuberous sclerosis complex). Their gene products form the TSC1-TSC2 complex (also named hamartin-tuberin complex) and, through its GAP (GTPase-activating protein) activity towards the small G-protein Rheb (Ras homolog enriched in brain), this complex is a negative regulator of mTORC1 (mammalian target of rapamycin complex 1) [13]. Because processes such as growth, autophagy and neuronal plasticity are known to be regulated, at least in part, through the mTOR pathway [14],[15] and because *Herc1^tbl^/Herc1^tbl^* mice show features related to these processes (neuronal degeneration and smaller size), we hypothesized that a mutant HERC1 protein might affect some of these events through deregulation of the mTOR pathway. With this guess in mind, we first analyzed whether the levels of the TSC1-TSC2 complex and mTOR protein were modified in the brain of *Herc1^tbl^/Herc1^tbl^* mice. We found that the levels of these proteins remained normal in these animals suggesting that the mutant HERC1 protein does not affect their stability (Figure 7B). We then checked whether the mTOR activity was modified, and to achieve this, we analyzed the phosphorylation of a substrate of the mTORC1 activity, the ribosomal protein S6 kinase 1 (S6K1). We observed a decrease of the phosphorylation of S6K1 at threonine 389 (P-T389-S6K1) in brain of *Herc1^tbl^/Herc1^tbl^* mutant mice (Figure 7C). These data were also confirmed and quantified in kidney where P-T389-S6K1 levels were found higher compared to +/+ mice (Figure 7C).

Because it had been previously reported that mTOR negatively regulates autophagy [16], we thought that the decrease of the mTORC1 kinase activity could correlate with an increase of autophagy in *tbl/tbl* mice. The conversion of the microtubule-associated protein light chain 3 (LC3-I) to its phosphatidylethanolamine-modified form (LC3-II) has been used as marker of the accumulation of autophagosomes [17]. We have measured by immunoblot analysis this autophagy marker observing an increase of LC3-II levels in brain and cerebellum of *tambaleante* mice (Figure 7B and 7C). To check that this increase was due to increases in autophagic activity and not to reduced turnover of autophagosomes [18], we also measured the steady-state levels of the known substrate for autophagy p62/SQSTM1 [19]. We observed a significant decrease in the steady-state levels of p62/SQSTM1 (Figure 7C) indicating that autophagic flux was not blocked. Altogether these data suggest that autophagy is induced in *tbl/tbl* mice. An attractive hypothesis would be that this activation is the cause of PC death in the *tambaleante* mice. This however is difficult to ascertain because an increase in autophagy is also a protective mechanism for cells in response to stressing stimuli. To determine whether this was the case or not, we analyzed the occurrence of autophagy at an earlier phase of the PC degeneration; in 2-month-old *Herc1^tbl^/Herc1^tbl^* cerebellum. Double labeling with antibodies against anti-calbindin D28-k and lysosome-associated membrane protein LAMP-1 allowed us to identify lysosomes with large cytoplasmic accumulations in dendrites and somata being particularly numerous in regions of the cerebellar cortex which had lost the calbindin D28-k expression (Figure 7E). Electron microscopy also performed on 2-month-old mice showed that PC bodies contained numerous autophagosomes (vacuoles with double membrane and filled with cytoplasmic organelles) and autolysosomes (vacuoles with a single membrane and filled with larger inclusions) (Figure 7F–7H). Altogether these data show that activation of autophagy in *Herc1^tbl^/Herc1^tbl^* mutant mice is indeed the earliest pathological process that seems to be involved in the degeneration and death of PCs. This high degree of autophagy is unique to the *Herc1^tbl^* mutation and has never been reported before for any other cerebellar mutation [20].

## Discussion

Genes of the *Herc/HERC* family are absent in prokaryotes and in eukaryotes such as fungi and plants. In mammalian genomes there are several *HERC* paralogous copies encoding two subgroups of proteins: large (HERC1–2) and small (HERC3–6 in human; HERC3–5 in mouse). The HERC1 giant protein, which was the first to be identified in a screening looking for human oncogenes, contains several domains (one HECT, two RLDs, seven WD40 repeats and one SPRY) involved in protein ubiquitilation, guanine nucleotide exchange and protein-protein or protein-lipid interaction. This structure probably reflects the multiple interactions of this protein with other cellular proteins. HERC1 interacts with phosphoinositides and with several other proteins such as clathrin, ADP-ribosylation factor (ARF), M2-pyruvate kinase and TSC2 and, through these interactions it has been involved in membrane trafficking, cell growth and proliferation [8], [10]–[12].

The Gly483Glu substitution that we found in *tambaleante* mice, which is located within the highly conserved RLD1 domain (Figure 3D–3E), presumably alters the structure of the HERC1 protein and very likely impairs its functions as well [10]–[11]. The structural alteration might be causative of an increase in its stability, leading to the observation of an unexpected increased level of this protein in all studied tissues (Figure 7A, 7B, and 7D). Impairment of HERC1 function through the mTOR pathway could explain the neuronal degeneration and the smaller size observed in *tambaleante* mice. Since mTOR has been reported to be a negative regulator of autophagy [16], a decrease of its activity would indeed correlate with an increase of autophagy observed in the PCs of *tambaleante* mice (Figure 7). Although future studies are required to understand the precise role of HERC1, we can however conclude that HERC1 has a profound impact in the animal growth and the maintenance of the cerebellum structure.

A consensus is emerging among molecular geneticists stressing that a missense mutation, affecting only one site of a multidomain protein, is sometimes of better value for gene annotation than a knockout allele that, in general, suppresses at once the protein. In the case of *Herc1^tbl^* the situation may be more complex. If the Gly483Glu amino acid substitution has an effect on the protein structure, then one may expect heterozygous mice to be affected to some extent. This however does not seem to be the case since, as far as we could observe from those *Herc1^tbl^/+* breeders that we kept for more than one year, we never noticed any symptoms in their gait or behaviour that would have been evocative of a pathology of the central nervous system (CNS). We did not conduct any observation at the histological level on these mice but it is not sure that this would have been of great value if we consider that a decrease in PCs number seems to be a common observation in mice heterozygous for most mutations affecting the cerebellum (*nr*, *Rora^sg^*, *Agtpbp1^pcd^* and *Reln^rl^*) [20]. It does not appear that the *Herc1^tbl^* mutation has a dominant negative effect on the HERC1 function because transgenic mice could rescue the *tambaleante* phenotype. Our data seem to indicate that the *tambaleante* protein is not functional or has acquired a different function to the wild-type protein and that the presence of wild-type protein has a dominant effect. For this reason, heterozygous or rescued mice, where the wild-type HERC1 protein is present, do not exhibit a *tambaleante* phenotype.

Because ataxia is the most apparent feature in *tambaleante* mice and because this symptom is commonly associated to a cerebellar defect, we focused more on this part of the CNS than on any other in our morphological survey. However, in all cases, the paraffin embedding and serial sectioning after Nissl staining that were achieved on adult mutant CNS, did not allow detection of any obvious lesion outside the cerebellum (retina was not analyzed). Nevertheless, the possibility that some type of alteration could be disclosed using more specialized methods (immunohistochemistry, electron microscopy) remains open.

In humans, genes encoding proteins with mutations in their RCC1 domains have been found to be involved in several diseases [11]. The best studied is probably the *RPGR* (Retinitis pigmentosa GTPase regulator) gene, which is responsible for 70–80% of the most severe forms of human the X-linked retinitis pigmentosa [16], and in which more than 200 independent mutations have been identified. Functional studies suggest a role for this protein in microtubule-dependent transport along cilia [21]. Another example is provided by the *ALS2* (amyotrophic lateral sclerosis 2-juvenile) locus, which encodes for a protein where mutations have been associated to an autosomal recessive form of juvenile-onset amyotrophic lateral sclerosis (jALS) [22],[23]. All mutations found in this gene lead to the production of truncated proteins. Interestingly, truncations affecting its amino-terminus, where the RCC1-like domain is located, lead to jALS with degeneration of upper and lower spinal cord moto**r**neurons, whereas less severe truncations in the protein Alsin lead only to degeneration of the upper moto**r**neurons [24],[25]. Gene expression of HERC1 has also been reported to be increased in human tumour cell lines [8] and decreased in heroin users with a genetic variation of the opioid receptor [26].

HERC2, the other member of the large HERC family, has also been found associated with pathologies. Mutations in the mouse *Herc2* gene were found to be responsible for the so-called *runty*, *jerky*, *sterile*-syndrome or, in short, *rjs*-syndrome, also known as *jdf-2* (*juvenile development and fertility-2*) [5],[6]. The pathogenic mechanisms of this syndrome are not known at the molecular level, but it has been suggested that at least some of its symptoms could be due to pituitary defects. In humans, the *HERC2* genomic locus, including several partially duplicated paralogs (duplicons) of *HERC2* [27],[28], corresponds to the chromosomal breakpoint region in deletions that cause the Prader-Willi and Angelman syndromes [29],[30] although lack of HERC2 protein does not seem to play a role in these syndromes [6]. Recently, it has been reported that a single nucleotide polymorphism in intron 86 of the *HERC2* gene determines human blue/brown eye colour by controlling the expression of the neighboring gene *OCA2* [31]–[33].

In summary, the present study unambiguously demonstrates that the gene *Herc1* is involved in the mutation *tambaleante* and shows, for the first time, that this gene has a profound impact on growth and maintenance of the cerebellar structure. To our knowledge, no other mutant allele has ever been reported at the *Herc1* locus before *Herc1^tbl^*. Considering the relative great size of this gene (78 exons - with a predicted coding region of 14,559 bp) this is rather surprising and probably means that a majority of the mutations likely to occur at this locus either have no deleterious effects or, most probably, that they are lethal *in utero* and accordingly remained undetected so far. This is an important difference with the *Herc2* locus where at least a dozen mutations have been reported that lead to the *rjs/jdf2* syndrome [5],[6]. This also means that, in spite of ancestral relationships, the two proteins have acquired some specific, non-redundant functions.

## Materials and Methods

### Animals

The *tambaleante* mutation is available at the RIKEN BioResource Center, Tsukuba, Japan (<http://www2.brc.riken.jp/> - Ref: RBRC00188). The mouse strain transgenic for the mouse BAC clone RP23-355L9 was generated in Kyoto University by direct *in ovo* injection. The mouse strain transgenic for the full-length human *HERC1* cDNA was generated at the Institute Pasteur. The transgene was previously generated in two steps: first, we digested pCIneo vector (The vector carries the cytomegalovirus (CMV) immediate-early enhancer/promoter, from Promega) with the restriction enzyme *BglII* and ligated the annealed oligonucleotide GATCTATCGATA generating a new *ClaI* restriction site in pCIneo vector. Then the full-length human *HERC1* cDNA [8] was cloned into this modified pCIneo (pJLR189). The transgene cassette was linearized by *Cla*I, purified by agarose gel and microinjected *in ovo*. All animal experiments were performed following European and Japanese institutional guidelines for animal handling and research.

### Genotyping

Tail DNA samples were prepared from mice according to [34] and PCR amplification of the exon containing the *tambaleante* mutation was performed using the primers: 5′-GCTTGTGGTAAAGGCAGCTATGGG-3′ and 5′-CCTCACATGTCCCCACACAC-3′, yielding a 476 bp product (PCR settings were: 94°C×5 minutes, 94°C×30 seconds, then annealing at 60°C for 30 seconds, and elongation at 72°C for 30 seconds. Number of cycles: 35). Amplification products were then digested with *Mbo*II enzyme and fractionated in a 10% PAGE to distinguish among *tbl/*+, *tbl/tbl* and *+/+* mice. Transgenic mice were PCR-genotyped using the primers: 5′-TGGTGGAAATAGTATCCCAC-3′ and 5′-CACGGTCAGTAGTCAGTGTC-3′, yielding a 588 bp product (PCR settings: 94°C×5 minutes, 94°C×30 seconds, then annealing at 55°C for 30 seconds, and elongation at 72°C for 45 seconds. Number of cycles: 35).

### RT–PCR

A mouse multiple-tissue and embryo cDNA set (Mouse MTC Panel I, Clontech) was used for expression analysis of *Herc1* gene. RT-PCR was performed with following primers: 5′-GAAGATGTGGATGCAGCAGA-3′ and 5′- GGTCTGTCCGGTGAAGGATA-3′ for mouse *Herc1* cDNA (199 bp), and *Gapdh* 5′ and 3′ PCR primers (983 bp) as a control. To assess transgene expression, total RNA was isolated from mouse brains using the Ultraspec RNA Isolation System (Biotecx). 2 µg of total RNA were reverse-transcribed using the cDNA Reverse Transcription kit (Applied Biosystems) and random primers. PCR was carried out with primers: 5′-AGTCGACTGGATCCGGTACC-3′ and 5′-AGTCTGGCAACTGTGGTCCT-3′ for the transgene and 5′-ATGGATGACGATATCGCTG-3′ and 5′-ATGAGGTAGTCCGTCAGGA-3′ for the actin control.

### Histology and immunohistochemistry

Mice were perfused transcardially with a fixative containing 4% formaldehyde in 0.1 M phosphate buffer after being deeply anesthetized by diethyl ether inhalation. The cerebellum was removed and postfixed in the same fixative for two hours, then embedded in gelatin 8% and subsequently processed for respective histological analyses. Tissue samples were stored at −80°C and cut on a cryostat. Cryosections were stained with H&E or immunostained with rabbit polyclonal anti-calbindin D28k or anti-LAMP-1 antibodies. For immunostaining, the cerebellum was processed according to [35].

### Neurobehavioural assays

The rotarod test was used to assess motor coordination and function (rotarod apparatus: ROTAROD/RS Panlab; diameter: 3.5 cm, length: 5 cm). For Figure 5, three groups were constituted: +/+ (n = 8, four males, four females), *tbl/tbl* (n = 9, four males, five females), *tbl/tbl Tg^HERC1cDNA^* (n = 5, two males, three females). Testing began at 4 weeks of age and was conducted until week 12 to follow the progression on each phenotype. In brief, animals were trained a constant speed (16 rpm) for 60 s. The animals were put on the rotarod until the latency to fall off reached the total time of 60 s. Each mouse was placed on the rotarod with its head in the direction of rotation and so had to turn to the opposite direction. We performed three trials per day with 2–6 min intervals, on three consecutive days. During the pauses between the turns, mice were allowed to rest in their home cages. After training, mice were evaluated once at 6, 8 and 12 weeks of age at a constant speed of 12 rpm until the latency of fall reached 1 min. The percentage (%) of animals that stayed for 60s is shown in Figure 5. For Figure 1, each mouse had three trials per test with an intertrial interval of 5 minutes. Mice (n = 4 to 9) were placed on the rotating drum at 20 rpm and the time the animal stayed on the rotarod without falling off was measured. The hind limb clasping reflex was assessed by holding the mouse by its tail for 30 seconds.

### Antibodies used

Horseradish peroxidase-coupled secondary antibodies (Molecular Probes); anti-mTOR and anti-P-T389-S6K1 (1A5) antibodies (Cell Signalling Technology); anti-TSC2 (C-20), anti-S6K1 (C-18) and anti-LAMP-1 (N-19) antibodies (Santa Cruz Biotechnology, Inc.); anti-CHC antibody (BD Transduction laboratories); anti-HERC1 antibodies [8]; anti-LC3 antibody (MBL); anti-calbindin D28k antibody (Swant, Bellinzona, Switzerland); anti-p62/SQSTM1 antibody (Abnova).

### Lysates and immunoblotting

Mice (3–7 months old) were euthanized by cervical dislocation. Organs were collected and frozen in liquid nitrogen and stored at −80°C until analysis. Tissues were prepared in lysis buffer (consisting of 10 mM Tris-HCl, pH 7.5, 100 mM NaCl, 1.5 mM MgCl_2_, 50 mM NaF, 1 mM sodium vanadate, 1 mM phenylmethylsulfonyl fluoride, 5 mg/ml leupeptin, 5 mg/ml aprotinin, 1 mg/ml pepstatin A, 50 mM β-glycerophosphate, 100 mg/ml benzamidine), homogenized in a motor-driven Polytron PT3000 (Kinematica AG) incubated in a precooled tube with CHAPS 0.3% for 20 minutes, and centrifuged at 13,500g for 15 min at 4°C. Total protein levels were measured by BCA (Pierce). Equal amounts of supernatant proteins (200µg/lane) were separated by electrophoresis. To analyze simultaneously in the same SDS/PAGE gel giant proteins such as HERC1 or mTOR and small proteins such as LC3, lysates were loaded in a combination of SDS/PAGE gels named LAG gel [36]. After running the gel overnight, the proteins were transferred to PVDF membranes and visualized by immunoblotting using specific antibodies as previously described [36]. Band intensities were analyzed with a gel documentation system (LAS-3000 Fujifilm). The protein levels were normalized with respect to CHC, mTOR or S6K1 levels and expressed as percentage of controls.

## Supporting Information

Figure S1 Rescue of the *tambaleante* phenotype with *Tg^RP23-355L9^*. Foot prints (A,B) and H&E staining of cerebellar sections (C,D) of *tbl/tbl* mice (A,C) and *tbl/tbl* mice transgenic for BAC RP23-355L9 at 12-weeks-old.
(0.34 MB TIF)

Figure S2 Expression of the transgene *Tg^HERC1cDNA^*. Total RNA was isolated from mouse brains (*tbl/tbl*; *Tg^HERC1cDNA^*/+; *tbl/tbl;Tg^HERC1cDNA^*/+). 2 µg of total RNA were reverse-transcribed using random primers. PCR was carried with specific primers for transgene and actin. Actin was used as control. Transgene cassette (pJLR189) was used as positive control and pDEST-HA vector as negative control. Amplified DNA was run in agarose gels stained with ethidium bromide.
(0.23 MB TIF)

Video S1 Tambaleante mouse. A 30-second video of a two-month-old *tambaleante* (*tbl/tbl*) male mouse.
(3.18 MB AVI)
